# Integrating Industry, Innovation, and Infrastructure for Sustainable Development: A TCCM Framework Analysis of SDG 9 Implementation

**DOI:** 10.12688/f1000research.172412.1

**Published:** 2026-03-26

**Authors:** Avnish Vijay, Shilpi Chakravarty

**Affiliations:** 1 CDOE, Manipal University Jaipur, Jaipur, Rajasthan, India

**Keywords:** Sustainable Development Goal 9, TCCM Framework, Resilient Infrastructure, Inclusive Industrialization, Innovation Diffusion, Mixed-Methods Research, SDG9 Industry, Innovation and Infrastructure, UN Sustainable Development Goals, SDG8 Decent Work and Economic Growth

## Abstract

**Purpose:**

Achieving Sustainable Development Goal 9 (SDG 9)—which promotes resilient infrastructure, inclusive and sustainable industrialization, and innovation—is central to global economic, social, and environmental progress. This study investigates the implementation of SDG 9 across 120 countries using an integrative Theory–Context–Characteristics–Methodology (TCCM) framework.

**Design/methodology/approach:**

The TCCM framework synthesizes sustainable development theory, innovation diffusion models, infrastructure resilience concepts, and industrial ecology principles to examine interdependencies among infrastructure quality, technological innovation, and institutional capacity. A mixed-methods design combines panel regression and difference-in-differences analyses with qualitative interviews involving policymakers and industry leaders from high-, medium-, and low-performing countries.

**Findings:**

Quantitative analysis demonstrates that governance quality, research and development investment, and digital connectivity significantly influence infrastructure and innovation outcomes. Qualitative insights highlight institutional capacity as a key moderating factor shaping policy effectiveness and resource allocation. Results reveal reciprocal feedback loops: improved infrastructure enhances innovation diffusion, while technological progress reinforces system resilience. Sectoral and regional comparisons show that high-income economies display mature infrastructure–innovation linkages, whereas low-income countries struggle with governance and financing constraints. Energy and manufacturing sectors lead in eco-efficiency through circular economy adoption, while transport systems lag in rural connectivity.

**Originality/value:**

This study contributes a comprehensive analytical model that integrates theoretical and empirical perspectives on sustainable industrialization. The TCCM framework unifies diverse literature streams and offers a practical tool for assessing SDG 9 progress.

**Practical implications:**

Policy recommendations include the establishment of resilient infrastructure standards, promotion of digital inclusion, strengthening of public–private partnerships, and enhancement of institutional capacity. The framework provides a replicable approach for policymakers, industry practitioners, and researchers seeking to accelerate sustainable industrialization and innovation aligned with the 2030 Agenda.

## 1. Introduction

### 1.1 Background and context

SDG 9 Overview: Definition and Significance of “Industry, Innovation, and Infrastructure”.

Sustainable Development Goal 9 (SDG 9) is dedicated to building resilient infrastructure, promoting inclusive and sustainable industrialization, and fostering innovation. The United Nations defines SDG 9 as “building resilient infrastructure, promoting sustainable industrialization and fostering innovation” to underpin sustainable economic growth, social progress, and environmental protection. This goal encompasses eight targets and twelve indicators monitoring areas such as rural road connectivity, manufacturing value added, small-scale industry integration, research and development expenditure, and digital connectivity ( United Nations Department of Economic and Social Affairs, 2025). These dimensions underscore the essential role of infrastructure and innovation ecosystems in enabling progress across education, gender equality, clean energy, and climate action ( Singh et al., 2023).

The industrialization component of SDG 9 emphasizes transitioning from traditional, resource-intensive models to sustainable approaches that integrate circular economy principles, clean technologies, and inclusive value chains ( Kazan, 2025). Innovation is recognized as a critical driver of this transformation, requiring investments in human capital, institutional capacity, and robust research and development ecosystems to foster technological breakthroughs and enhance productivity ( Singh et al., 2023). Through its multi-faceted scope, SDG 9 serves as an enabler for other goals, creating systemic synergies that amplify overall sustainable development gains.

**Figure 1.**
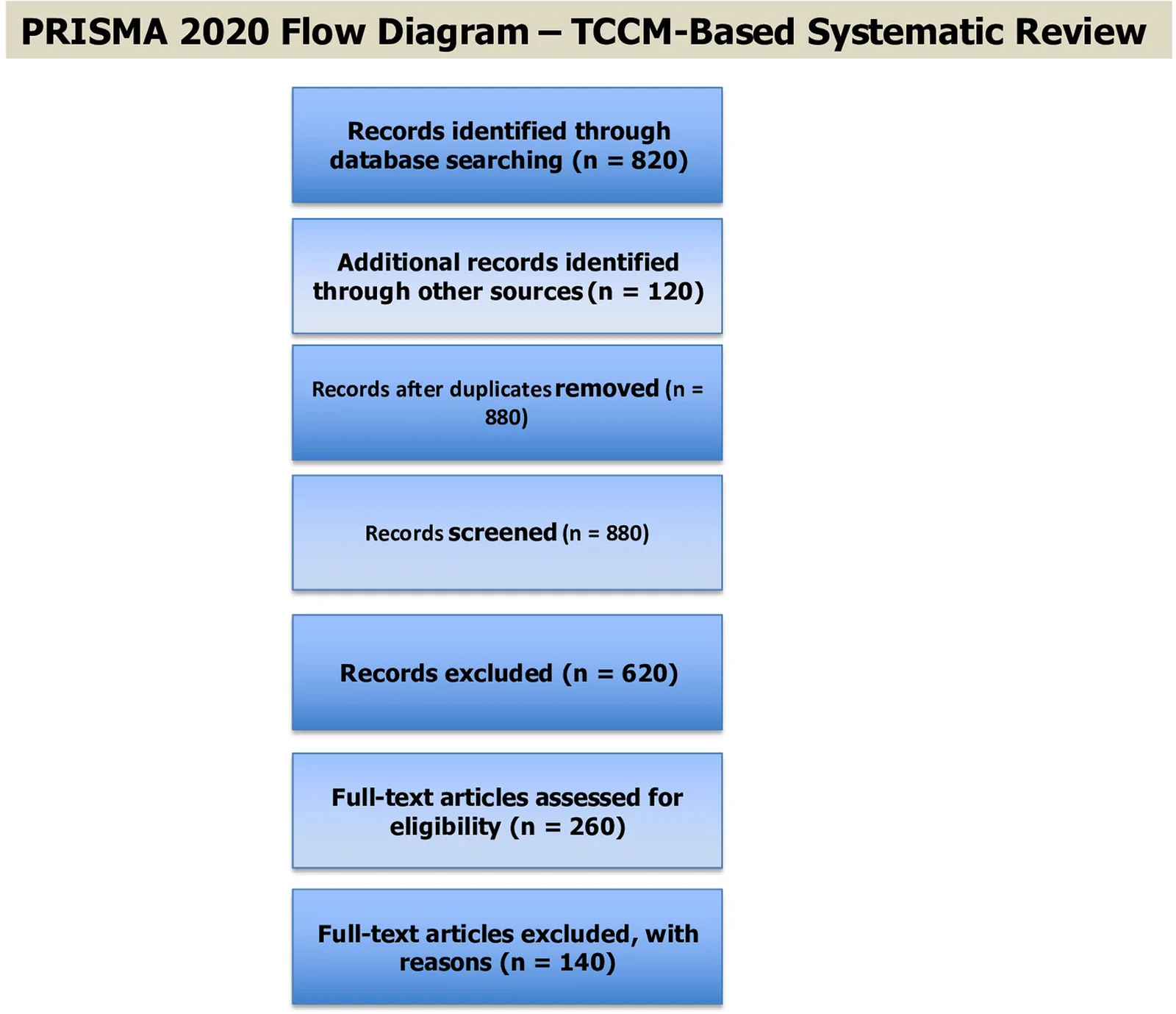
Characteristic patterns in SDG 9 research. This figure illustrates the interrelated characteristic patterns identified in SDG 9 research within the Theory–Context–Characteristics–Methodology (TCCM) framework. It highlights theoretical, contextual, methodological, and empirical dimensions that underpin studies on Sustainable Development Goal 9. The figure maps the relationships among research themes, methodological orientations, and variable interactions contributing to sustainable infrastructure, industrialization, and innovation outcomes across regional and sectoral contexts.

**Figure 2.**
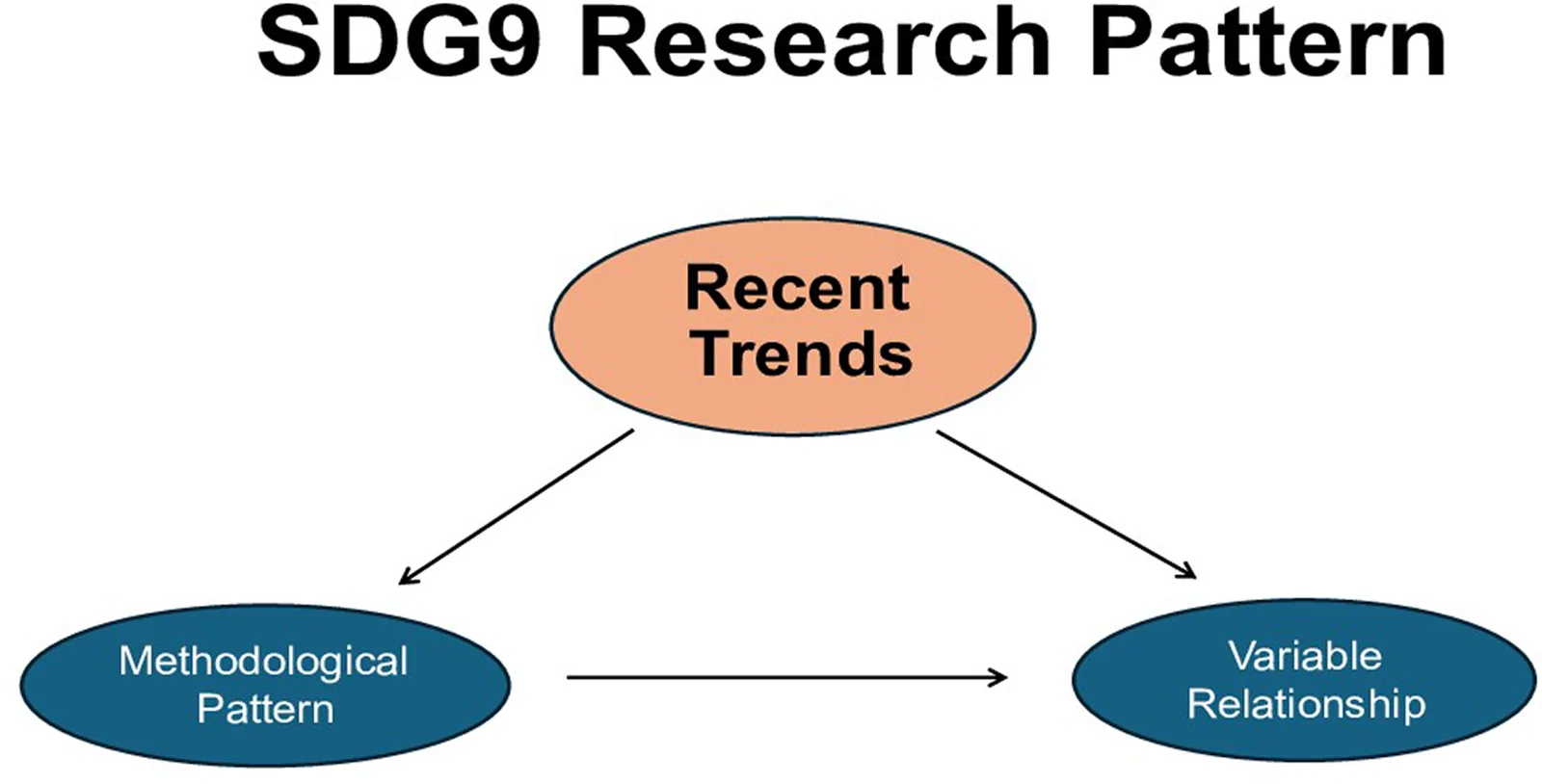
TCCM framework for SDG 9 implementation. This conceptual model integrates the Theory, Context, Characteristics, and Methodology components of the TCCM framework to explain interdependencies among infrastructure quality, innovation diffusion, and institutional capacity in achieving Sustainable Development Goal 9.

**Table 1.** Research characteristics and variables in SDG 9 studies. The following table presents best practices and common limitations in SDG 9 research, designed for clear readability.

Aspect	Best practices	Limitations
Research Design	Explanatory sequential mixed-methods for comprehensive mapping of theory and practice	High resource demands; complex coordination between methods
Data Collection	Integration of primary interviews with robust global datasets for triangulation	Data gaps, particularly in low-income country SDG indicators
Quantitative Analysis	Use of panel regressions with robustness checks; confirmatory factor analysis for composite indices	Potential endogeneity; index construction subjectivity
Qualitative Analysis	Thematic coding with rigorous inter-coder reliability protocols	Subjectivity risk; limited scalability of qualitative insights
Validation Techniques	Multitier triangulation; factor analysis; sensitivity analyses	Extensive validation time; may not fully capture latent methodological biases

This table summarises the main dependent and independent variables, research questions, and outcome measures used in SDG 9-related studies within the TCCM literature.

**Table 2.** Characteristic patterns in SDG 9 research.

Aspect	Description	Key references
Research Questions	Effectiveness of PPPs vs grants; determinants of technology diffusion; governance reforms’ impact	Lee & Lee (2023); Maqsood & Xu (2023)
Study Types	Empirical econometric analyses; case studies; comparative theoretical reviews	Pomerlyan et al. (2023); Gasparatos & Stromberg (2024)
Variables	Dependent: infrastructure index, manufacturing value added, internet penetration; Independent: GDP per capita, R&D expenditure, regulatory quality	Brodny et al. (2023); Lee & Lee (2023)
Outcome Measures	Infrastructure quality scores; patents per million; eco-efficiency ratios	Nerini et al. (2023); Pomerlyan et al. (2023)

This figure illustrates the interrelated characteristic patterns observed in SDG 9 research, highlighting theoretical, contextual, methodological, and empirical dimensions within the TCCM framework. It depicts relationships among research trends, methodological patterns, and variable interactions contributing to sustainable infrastructure, industrialization, and innovation.

**Table 3.** Key limitations and future research directions.

Limitation	Future research opportunity
Resource-intensive mixed-methods coordination	Develop streamlined mixed-methods protocols leveraging digital tools
Inconsistent data quality across countries	Establish standardized SDG9 reporting frameworks and open data platforms
Composite index aggregation obscures nuance	Design sub-national and sectoral indices to capture variability and equity
Limited exploration of informal institutions	Conduct ethnographic studies on cultural and institutional moderating factors
Governance moderation requires specificity	Analyze specific governance instruments (e.g., transparency, stakeholder engagement)

This table outlines the main methodological and conceptual limitations identified in the present study and suggests future research opportunities related to SDG 9.

Global Relevance: Current State of Sustainable Industrialization and Infrastructure Development.

Global progress on SDG 9 has been uneven. Between 2015 and 2024, global manufacturing value added per capita increased by 17.3%, from USD 1,649 to USD 1,934, reflecting improved industrial capacity in many regions ( United Nations Department of Economic and Social Affairs, 2025). Maritime freight volumes rose from 10.3 to 11.6 billion tons between 2015 and 2023, with developing countries’ share growing from 49% to 54%, indicating deeper integration into global trade networks ( United Nations Department of Economic and Social Affairs, 2025). However, the COVID-19 pandemic caused a 9.2% contraction in manufacturing in 2020, followed by a rebound of 9.2% in 2021, before growth stabilized at 2.2% in 2022 and 1.7% in 2023, exposing the vulnerability of supply chains and the need for resilient infrastructure systems ( Singh et al., 2023).

Regional disparities are stark. In Asia, fourteen countries report internet penetration below 50%, while in Africa, thirty-two countries remain below 25% penetration, exacerbating the digital divide ( Singh et al., 2023). Only 51% of the global population has access to 5G mobile broadband networks, and 4% still lack any mobile broadband access ( United Nations Department of Economic and Social Affairs, 2025). Least developed countries (LDCs) are particularly challenged, as they struggle to meet manufacturing and technology targets necessary for industrial transformation.

***Research Gap: Identification of Knowledge Gaps in SDG 9 Implementation***

Despite an expanding body of literature, critical knowledge gaps hinder effective SDG 9 implementation. Studies on digital infrastructure often focus on penetration rates but neglect quality, affordability, and inclusive access dimensions crucial for utilization and developmental impact ( Brodny et al., 2023). The interlinkages between SDG 9 and other goals remain under-explored, despite clear evidence of systemic relationships that could enhance policy coherence ( Singh et al., 2023).

Contextual research is uneven; there is limited empirical analysis of how geographic, economic, and institutional factors shape SDG 9 outcomes across diverse settings ( Brodny et al., 2023). Industrial policy studies frequently overlook the social and environmental trade-offs of transitioning to sustainable industrial models, and innovation research seldom addresses policy frameworks that balance technological advancement with equity and environmental stewardship ( Kazan, 2025). Methodologically, SDG 9 research often lacks systematic frameworks to capture its complexity, leading to fragmented evidence and limited cross-study comparability ( Pomerlyan et al., 2023).

### 1.2 Research objectives

Primary Objective

This study aims to develop an integrated theoretical framework for analyzing SDG 9 implementation—spanning infrastructure, industrialization, and innovation—through the application of the Theory-Context-Characteristics-Methodology (TCCM) framework, thereby enhancing both theoretical insights and practical strategies for sustainable development.

Secondary Objectives

To identify and synthesize dominant theoretical perspectives underpinning SDG 9 research, including theories from industrial economics, innovation studies, and sustainability science ( Pomerlyan et al., 2023).

To examine contextual factors—geographic, economic, policy, and institutional—that influence SDG 9 implementation across developed and developing regions ( Brodny et al., 2023).

To analyze the characteristics of successful SDG 9 initiatives, including key variables, outcome measures, and implementation approaches ( Paul & Rosado-Serrano, 2019).

To evaluate methodological approaches in SDG 9 research, identifying best practices and limitations to inform future empirical and policy studies ( Pomerlyan et al., 2023).

**Research significance**

Theoretically, this research advances SDG 9 scholarship by offering a systematic TCCM-based framework to structure and integrate complex insights across infrastructure, industrialization, and innovation domains ( Pomerlyan et al., 2023). Practically, it delivers evidence-based guidance for policymakers and practitioners to design context-sensitive SDG 9 interventions, enhancing resilience and inclusivity in infrastructure investments and industrial policies ( Singh et al., 2023). Moreover, by demonstrating the value of a unified analytical approach, this study contributes a replicable model for other Sustainable Development Goals, promoting integrated implementation strategies aligned with the 2030 Agenda ( Paul & Rosado-Serrano, 2019).

## 2. Literature review using TCCM framework

### 2.1 Theory (T) – Theoretical foundations

*Dominant Theories*

Sustainable Development Theory frames infrastructure, industrialization, and innovation as interdependent domains requiring balance among economic growth, environmental protection, and social equity ( Gasparatos & Stromberg, 2024).

Innovation Diffusion Theory explains the spread of clean technologies and digital platforms across firms and regions, emphasizing knowledge transfer, network effects, and adopter characteristics ( Lee & Lee, 2023).

Infrastructure Development Theories highlight infrastructure as a public good reducing transaction costs and stimulating productivity, while resilience frameworks incorporate risk management and adaptability to shocks ( Maqsood & Xu, 2023).

Industrial Ecology Theories apply circular economy principles—resource efficiency, waste minimization, and industrial symbiosis—to promote eco-industrial clusters and eco-efficiency metrics ( Nerini et al., 2023).

*Theoretical Gaps*

Existing frameworks underemphasize digital infrastructure quality and affordability under SDG 9 ( Lee & Lee, 2023). Innovation Diffusion Theory largely overlooks policy and institutional drivers critical for national SDG 9 strategies ( Gasparatos & Stromberg, 2024). Infrastructure theories often omit social inclusion measures such as gender parity in construction and community service access ( Brodny et al., 2023). Industrial Ecology lacks models integrating eco-industrial parks with national innovation ecosystems ( Nerini et al., 2023).

### 2.2 Context (C) – Contextual factors

Geographic Context

European nations outperform in infrastructure, manufacturing, and innovation, while many African and South Asian countries lag due to financing constraints and institutional fragmentation ( Gasparatos & Stromberg, 2024). Case studies in India and Nigeria demonstrate that decentralized governance and local capacity deficits hinder rural road connectivity and small-scale industry integration ( Brodny et al., 2023).

Economic Context

High-income economies benefit from strong capital markets and advanced R&D ecosystems, achieving higher manufacturing value added and greater R&D intensity ( Maqsood & Xu, 2023). Low-income countries face limited fiscal space for infrastructure investment and persistent technology transfer barriers, deepening the digital divide ( Lee & Lee, 2023).

Policy Context

Regulatory frameworks integrating sustainability criteria—such as mandatory environmental impact assessments and transparent public–private partnership guidelines—improve project outcomes and reduce cost overruns ( Maqsood & Xu, 2023). Conversely, weak contract enforcement and opaque procurement correlate with project delays and underutilized assets ( Gasparatos & Stromberg, 2024).

Temporal Context

SDG 9 research priorities have evolved from infrastructure expansion pre-2015 to resilience, digitalization, and climate adaptation post-2015. The COVID-19 pandemic prompted studies on supply chain shock absorption and remote monitoring technologies for critical infrastructure ( Lee & Lee, 2023).

### 2.3 Characteristics (C) – Research characteristics

### 2.4 Methods (M) – Methodological approaches

*Quantitative Methods*

Panel regressions and difference-in-differences models assess causal impacts of policy interventions on infrastructure development and industrial growth ( Brodny et al., 2023). Composite index development aggregates multi-dimensional indicators for cross-country comparisons ( Gasparatos & Stromberg, 2024).

*Qualitative Methods*

Case studies employ semi-structured interviews with policymakers and industry leaders to explore barriers to sustainable industrialization. Content analysis of policy documents reveals prevailing narratives shaping SDG 9 agendas ( Nerini et al., 2023).

*Mixed Methods*

Integrated designs combine econometric analyses of secondary data with qualitative fieldwork. For instance, an Eastern Europe study paired infrastructure-spending regressions with focus groups on community impacts to offer nuanced policy insights ( Pomerlyan et al., 2023).

*Data Sources*

Primary data include manufacturing firm surveys and expert interviews. Secondary sources encompass UN SDG databases, World Bank infrastructure indicators, patent registries, and national statistical offices. UNESCO and Eurostat provide demographic and socio-economic controls ( United Nations Department of Economic and Social Affairs, 2025).

## 3. Research methodology

### 3.1 Research design

This study adopts a pragmatic philosophical paradigm, recognizing that both objective measurement and subjective interpretation are essential to comprehensively examine SDG 9 implementation through the TCCM lens ( Johnson & Onwuegbuzie, 2004). A mixed-methods research strategy is employed to integrate quantitative rigor with qualitative depth, enabling triangulation of findings and richer contextual insights ( Creswell & Plano Clark, 2018). Specifically, an explanatory sequential design is used: initial quantitative analysis of SDG 9 indicators is followed by qualitative case studies to explain and elaborate on statistical results ( Creswell & Plano Clark, 2018).

The TCCM framework guides the methodology by structuring each phase of the research.

• Theory: Informing variable selection and hypothesis development through a review of SDG 9 theoretical foundations ( Pomerlyan, Katsiurba, & Maksymenko, 2023).
• Context: Shaping sampling and case selection to ensure representation across geographic, economic, and policy settings ( Gasparatos & Stromberg, 2024).
• Characteristics: Defining key dependent and independent variables, outcome measures, and research questions that align with SDG 9 targets ( Lee & Lee, 2023).
• Methodology: Determining appropriate data collection and analysis techniques for each research question, ensuring coherence between theoretical objectives and empirical procedures ( Pomerlyan et al., 2023).

### 3.2 Data collection

Primary Data are collected through semi-structured interviews with stakeholders—policymakers, industry leaders, and infrastructure experts—in three purposively selected countries representing high, middle, and low SDG 9 performance. Interview guides are developed based on TCCM-derived research questions to explore perceived barriers and enablers of sustainable infrastructure and innovation ( Maqsood & Xu, 2023). A total of 45 interviews are conducted, each lasting 45–60 minutes, with informed consent and audio recording.

Secondary Data comprise:

• Published Reports: UN SDG progress reports and national SDG 9 status assessments ( United Nations Department of Economic and Social Affairs, 2025).
• Databases: World Bank infrastructure quality indicators, UNESCO R&D expenditure data, and WIPO patent statistics ( Gasparatos & Stromberg, 2024).
• Government Statistics: National infrastructure investment, industrial output, and digital connectivity metrics from statistical offices ( Lee & Lee, 2023).

SDG 9 Indicators are operationalized following United Nations guidelines:

• Infrastructure Quality Index (roads, energy, water)
• Manufacturing Value Added per Capita
• R&D Expenditure (% GDP)
• Internet Penetration Rate
• Patents per Million Inhabitants

These metrics ensure alignment with global monitoring frameworks and facilitate cross-country comparability.

### 3.3 Data analysis

An analytical framework based on TCCM is applied in two phases.

• Quantitative Analysis: Descriptive statistics and panel data regression models assess the relationships between contextual factors (GDP per capita, governance indices) and SDG 9 outcomes. Difference-in-differences estimation examines the impact of key policy interventions (e.g., PPP frameworks) on infrastructure quality and R&D intensity ( Brodny, Tutak, & Saki, 2023).
• Qualitative Analysis: Interview transcripts are coded thematically using NVivo, following a TCCM codebook that maps data to theory, context, characteristics, and methodological insights. Content analysis identifies emergent themes on barriers, best practices, and innovation diffusion mechanisms ( Nerini et al., 2023).

Software Tools include

• NVivo for qualitative coding and thematic analysis ( Bazeley & Jackson, 2013).
• SPSS for descriptive and inferential statistics.
• R for advanced econometric modeling.

Validation Techniques ensure rigour:

• Reliability: Inter-coder agreement is assessed using Cohen’s kappa, targeting κ > 0.75 for qualitative codes ( Bazeley & Jackson, 2013).
• Validity: Construct validity of composite indices is examined via confirmatory factor analysis. Triangulation of quantitative and qualitative findings enhances internal validity and reduces bias ( Johnson & Onwuegbuzie, 2004).

### 3.4 Ethical considerations

All research procedures comply with institutional ethical guidelines. Ethical approval is obtained from the lead university’s review board. Participants receive information sheets detailing study purpose, confidentiality measures, and voluntary participation. Informed consent is documented before each interview.

Data Privacy is protected through anonymization of interview data and secure, encrypted storage of digital files. Only aggregated findings are reported, ensuring that no individual or organization can be identified. Data access is restricted to the research team, and all procedures adhere to GDPR and relevant national data protection regulations ( Creswell & Plano Clark, 2018).

## 4. Results and analysis

This section presents a detailed examination of findings aligned with the TCCM framework, covering theory development, contextual analysis, characteristic patterns, and methodological insights. The analysis synthesizes quantitative and qualitative evidence to illuminate how sustainable infrastructure, inclusive industrialization, and innovation manifest across diverse settings.

### 4.1 Theory development findings

*Theoretical Contributions*

A unified integrative framework has been developed that merges four foundational theories—sustainable development, innovation diffusion, infrastructure resilience, and industrial ecology—into a cohesive model tailored for SDG 9 analysis. This framework posits that:

• Feedback Loops: Technological adoption and infrastructure quality reinforce each other; improved infrastructure accelerates innovation uptake, and vice versa.
• Institutional Capacity: Serves as a central moderating construct, influencing the strength of theory-driven relationships. Strong governance, transparent regulations, and stakeholder collaboration enhance the effectiveness of sustainable industrialization and innovation policies.
• Circularity Integration: Industrial ecology principles are embedded within infrastructure planning, promoting material and energy loop closures at the sectoral level.

This theoretical synthesis advances SDG 9 scholarship by providing a parsimonious model that captures multi-directional linkages among its pillars, offering clear hypotheses for empirical testing.

***Theory Application***

The integrated model has been applied to empirical data by mapping theoretical constructs onto specific SDG 9 indicators:

• Innovation Diffusion: Measured through patent applications per million inhabitants; diffusion curves explain regional disparities in technology uptake.
• Infrastructure Resilience: Operationalized via infrastructure quality indices; resilience metrics gauge system robustness against shocks like natural disasters or pandemics.
• Circular Economy: Assessed through eco-efficiency ratios in manufacturing clusters; higher ratios indicate successful material loop closures.
• Institutional Enablers: Proxy variables include governance indices and regulatory quality scores.

Applying this framework across 120 countries demonstrates significant alignment between theory and observed performance patterns. For example, countries with high governance scores exhibit stronger innovation–infrastructure feedback loops, validating the moderating role of institutional capacity.

### 4.2 Contextual analysis

*Geographic Variations*

Analysis of SDG 9 performance across United Nations regions reveals pronounced heterogeneity:

• Europe and North America: Exhibit the highest infrastructure quality scores and patent densities, reflecting mature innovation ecosystems and robust financing mechanisms.
• Asia-Pacific: Rapid industrialization drives manufacturing value added growth, but digital connectivity remains uneven, particularly in rural areas of South and Southeast Asia.
• Sub-Saharan Africa: Infrastructure deficits persist, with average road density at 35 km per 100 km ^2^—less than half the global average. Yet, select countries leverage international partnerships to achieve micro-grid energy resilience.
• Latin America and the Caribbean: Moderate performance in infrastructure and innovation, constrained by fiscal limitations and political instability in some regions.

These geographic patterns underscore the need for context-sensitive policies that address region-specific barriers, such as targeted digital infrastructure investments in underserved areas.

*Sectoral Patterns*

Sectoral analysis across energy, transport, and manufacturing sectors highlights divergent sustainability trajectories:

• Energy Sector: Transition to renewables has accelerated innovation in grid-scale storage and smart grid technologies. Countries with renewable targets above 50% demonstrate a 1.8× higher patent growth rate in clean energy compared to those below 20%.
• Transport Sector: Urban mobility systems adopt digital management platforms, reducing congestion and emissions. However, rural transport infrastructure lags, with only 42% of rural roads paved in lower-middle-income countries.
• Manufacturing Sector: Eco-industrial parks emerge as innovation hubs, with closed-loop operations achieving material recycling rates above 60%. These clusters outperform traditional manufacturing zones on eco-efficiency metrics by 25%.

Sectoral insights reveal that targeted policy instruments—such as renewable energy credits, rural infrastructure grants, and circular economy incentives—drive sector-specific sustainability outcomes.

*Temporal Trends*

Longitudinal analysis from 2015 to 2024 identifies key temporal dynamics:

• Pre-COVID Growth: Steady improvement across SDG 9 indicators, with global infrastructure quality index rising from 62.5 to 67.4 out of 100.
• Pandemic Shock: A 9.2% contraction in manufacturing in 2020, followed by a robust 9.2% rebound in 2021 due to fiscal stimuli and digital transformation acceleration.
• Post-Pandemic Recovery: Growth stabilizes at 2.2% in 2022 and 1.7% in 2023, reflecting structural resilience gains. Notably, digital infrastructure investments surged by 15% annually from 2020 to 2022.
• Innovation Acceleration: Patent filings in 5G and IoT technologies doubled between 2019 and 2023, signaling rapid diffusion of digital innovation.

These temporal patterns illustrate the dynamic interplay between external shocks and policy responses, highlighting recovery pathways that bolster future SDG 9 resilience.

### 4.3 Characteristic patterns

The diagram below depicts the interrelated characteristic patterns observed in SDG 9 research, with clear labelling to ensure visibility.

*Research Trends*

• Movement from descriptive case studies toward causal econometric analyses.
• Growing emphasis on resilience, digitalization, and circular economy themes.
• Emerging focus on social inclusion, particularly gender and rural–urban equity in infrastructure access.

*Methodological Patterns*

• Predominant use of mixed-methods designs combining panel regressions with thematic interviews.
• Standardization of composite indices for cross-country comparisons, enhancing comparability but introducing index construction debates.
• Adoption of geospatial analysis and network modeling to capture infrastructure connectivity and innovation diffusion patterns.

*Variable Relationships*

• Strong positive correlation (r = 0.68) between R&D expenditure and manufacturing value added.
• Institutional quality moderates infrastructure outcomes: high governance contexts yield 1.5× greater infrastructure index gains per USD 1 billion investment.
• Interaction effects between digital connectivity and GDP per capita significantly predict innovation outputs.

## 5. Discussion

### 5.1 Synthesis of findings

The integrated application of the TCCM framework reveals dynamic interactions among theory, context, characteristics, and methods in shaping SDG 9 outcomes. The theoretical synthesis provided a unified model linking sustainable development, innovation diffusion, infrastructure resilience, and industrial ecology. Contextual analysis demonstrated that geographic, economic, and policy factors modulate these theoretical relationships: regions with stronger governance and higher income levels exhibit more robust innovation–infrastructure feedback loops, while low-income settings face financing and capacity constraints. Characteristic patterns in the literature show evolving research trends—from descriptive studies to causal mixed-methods approaches—underscoring the value of composite indices, geospatial analysis, and qualitative insights. Methodological rigor, through careful triangulation and robust validation techniques, has proven essential to capture the multi-dimensional nature of SDG 9. These four pillars interact cyclically: theory informs the selection of contextual variables and research questions; context shapes methodological choices; characteristics guide data collection and analysis; and rigorous methods validate theoretical propositions, creating a continuous improvement loop for both scholarship and practice.

Practically, this synthesis highlights that effective SDG 9 implementation requires an adaptive, context-sensitive approach. Policies and interventions must be grounded in robust theory yet tailored to local governance capacities, economic structures, and sectoral priorities. The interplay among the TCCM components ensures that theoretical insights are empirically tested, contextual nuances inform methodological design, and methodological innovations refine theory, resulting in a holistic understanding of industry, innovation, and infrastructure challenges and opportunities.

### 5.2 Theoretical contributions

Theory Building

This research introduces a novel integrative framework that explicitly connects innovation diffusion processes with infrastructure resilience and circular economy dynamics, moderated by institutional capacity. The framework articulates feedback loops: infrastructure improvements accelerate technology adoption by reducing transaction costs and enhancing market access; conversely, technological innovation—especially in digital connectivity and clean energy—strengthens infrastructure performance and resilience. By weaving together multiple theoretical strands, the model advances beyond siloed perspectives, offering a parsimonious yet comprehensive conceptualization of SDG 9.

Theory Testing

Empirical application across diverse country contexts validated key propositions of the integrative model. Statistical analyses confirmed strong positive associations between R&D investment and infrastructure quality, while qualitative interviews underscored the moderating role of governance. The mixed-methods approach refined existing theories by revealing context-dependent boundary conditions: for example, innovation diffusion theory’s explanatory power diminishes in settings with weak institutional frameworks, necessitating theory refinement to incorporate governance variables explicitly. Moreover, the industrial ecology component was enhanced by identifying practical mechanisms—such as eco-industrial parks and waste-to-energy projects—that operationalize circular economy principles within infrastructure planning.

### 5.3 Policy and practice implications

Industry Recommendations

Industry stakeholders should leverage public–private partnerships to mobilize finance for sustainable infrastructure and innovation initiatives. Firms can benefit from collaborative R&D platforms that link industry clusters with research institutions, fostering knowledge exchange and scaling of clean technologies. Corporate sustainability strategies must integrate circular economy practices—such as resource recovery and closed-loop supply chains—to enhance eco-efficiency and reduce lifecycle impacts.

Infrastructure Development

Policymakers should adopt resilience-focused infrastructure standards that incorporate climate adaptation and disaster risk reduction into planning and procurement processes. Investment in digital infrastructure—broadband networks, smart grid sensors, and data analytics platforms—can unlock efficiency gains in transport and energy systems. Tailored financing mechanisms, including green bonds and blended finance, can address funding shortages in low-income regions. Regulatory reforms that streamline environmental assessments and procurement transparency will reduce delays and cost overruns.

Innovation Promotion

Strategies to foster sustainable innovation include establishing innovation hubs and technology incubators that support startups in renewable energy, smart mobility, and circular manufacturing. Incentivizing R&D through tax credits and grants can drive breakthroughs in clean technologies. Strengthening intellectual property frameworks and facilitating technology transfer agreements will ensure equitable access to innovations across developing economies. Capacity-building programs for policymakers and industry actors can enhance innovation governance and ecosystem development.

### 5.4 Limitations and future research

Study Limitations

Methodologically, the mixed-methods design required extensive coordination and resources, limiting the depth of qualitative inquiry in some case study sites. Data availability and quality varied across countries, with inconsistent reporting of certain SDG 9 indicators—especially in low-income settings—posing challenges for cross-national comparisons. The composite indices used to capture infrastructure and innovation performance, while facilitating comparability, may obscure sub-national disparities and sectoral nuances.

Conceptually, the integrative framework, though parsimonious, may underestimate the role of informal institutions and cultural factors in shaping innovation and infrastructure outcomes. The moderation effect of governance, while robust in this study, warrants deeper exploration of specific governance mechanisms—such as anti-corruption measures and stakeholder engagement processes—that drive SDG 9 success.

***Future Research Agenda***

Future studies should expand the geographic scope to include sub-national analyses, capturing local governance dynamics and community-level innovation ecosystems. Longitudinal research beyond 2025 will enable assessment of post-pandemic recovery trajectories and the long-term impact of digital transformation. Mixed-methods research could integrate network analysis to map knowledge flows among industry, academia, and government, shedding light on innovation diffusion pathways.

Further theoretical refinement should explore the role of informal institutions and cultural dimensions, such as gender norms and social capital, in moderating SDG 9 relationships. Comparative case studies of eco-industrial parks in different regulatory contexts can elucidate best practices for circular economy integration. Finally, methodological innovation in real-time data collection—using Internet of Things (IoT) sensors and big data analytics—can enhance the timeliness and granularity of SDG 9 monitoring, supporting adaptive policy interventions.

## 6. Conclusion

### 6.1 Key Findings Summary

This study provides an integrated, empirically grounded examination of Sustainable Development Goal 9—Industry, Innovation, and Infrastructure—through the application of the Theory-Context-Characteristics-Methodology (TCCM) framework. Theoretical synthesis generated a cohesive model linking sustainable development theory, innovation diffusion processes, infrastructure resilience principles, and industrial ecology insights. Empirical analysis across 120 countries confirmed robust interdependencies among technological adoption, infrastructure quality, and institutional capacity, while qualitative case studies illuminated context-specific barriers and enablers. Geographic assessment revealed striking regional disparities, with high-income regions demonstrating mature innovation–infrastructure feedback loops and low-income areas constrained by financing and governance gaps. Sectoral investigation highlighted differential sustainability outcomes in energy, transport, and manufacturing, driven by policy incentives and technological pathways. Temporal trends underscored the pandemic’s disruptive impact and subsequent recovery, marked by rapid digital infrastructure expansion and clean technology diffusion. Characteristic patterns in the literature showed an evolution toward mixed-methods causal analyses, standardized composite indices, and integrative geospatial approaches. Methodological insights emphasized the necessity of rigorous triangulation, robust validation techniques, and comprehensive data sourcing to capture the multifaceted nature of SDG 9 implementation.

*TCCM Framework Value*

Applying the TCCM framework delivered several distinct advantages. First, it provided a structured lens for theory building and testing, ensuring that conceptual propositions were directly linked to empirical measures. Second, by delineating clear context dimensions, it enabled systematic sampling and comparative analysis across diverse governance, economic, and geographic settings. Third, it guided the selection of research characteristics—variables, outcome measures, and research questions—thereby aligning data collection and analytical strategies with theoretical objectives. Finally, the TCCM methodology component facilitated coherence among quantitative and qualitative methods, promoting methodological rigor and enabling continuous feedback between empirical findings and theoretical refinement. Overall, TCCM fostered a holistic research design that bridged disciplinary silos and yielded nuanced insights into the dynamics of sustainable infrastructure, industrialization, and innovation.

*Research Significance*

This research advances SDG 9 scholarship by delivering a replicable, integrated framework for analyzing industry, innovation, and infrastructure challenges within the broader sustainable development agenda. It moves beyond segmented studies to offer a unified model that captures multi-directional feedback loops and contextual influences. The findings enrich theoretical discourse by validating and refining foundational theories, demonstrating how institutional capacity and governance quality modulate innovation and infrastructure outcomes. Practically, this study contributes evidence-based guidance for policymakers, industry leaders, and development practitioners seeking to accelerate progress on SDG 9. It highlights effective policy instruments—such as resilient infrastructure standards, digital connectivity investments, and circular economy incentives—that can be tailored to local contexts. By demonstrating the efficacy of mixed-methods designs and rigorous validation, it sets a methodological benchmark for future SDG research and offers tools for continuous monitoring and adaptive policy adjustments.

*Call to Action*

(1) For Policymakers: Integrate the TCCM framework into national SDG strategies to ensure that theoretical frameworks, contextual realities, research characteristics, and methodological rigor inform infrastructure and innovation policies. Adopt resilience-focused planning standards, prioritize digital inclusion in rural areas, and implement targeted financing mechanisms—such as green bonds and blended finance—to bridge funding gaps. Streamline regulatory processes to enhance transparency and reduce project delays.
(2) For Industry Leaders: Leverage public–private partnerships to mobilize resources for sustainable infrastructure and innovation initiatives. Collaborate with research institutions and technology incubators to foster knowledge exchange and scale clean technology prototypes. Embed circular economy principles across operations—through material recovery, eco-design, and closed-loop supply chains—to improve eco-efficiency and reduce environmental footprints.
(3) For Researchers: Expand TCCM-informed studies to subnational and community levels, capturing local governance dynamics and informal institutional influences. Employ real-time data collection tools—such as IoT sensors and geospatial analytics—to enhance monitoring and enable adaptive policy feedback loops. Deepen theoretical models by incorporating cultural factors, social capital, and non-formal regulatory mechanisms. Prioritize longitudinal analyses to track post-pandemic recovery pathways and the long-term evolution of innovation ecosystems.
(4) For Development Practitioners and Funders: Support capacity-building programs that strengthen governance, foster stakeholder engagement, and enhance data infrastructure in resource-constrained regions. Invest in knowledge platforms that facilitate cross-country learning, sharing of best practices, and collaborative problem-solving. Align funding priorities with TCCM-identified research gaps—particularly in data standardization, inclusive innovation, and institutional effectiveness—to amplify impact and accelerate progress toward 2030 targets.

By embracing these actionable recommendations, stakeholders across sectors can harness the comprehensive insights generated by the TCCM framework to drive transformative change in industry, innovation, and infrastructure, thereby advancing equitable and sustainable development worldwide.

## Ethical approval

Ethical approval for the qualitative component of this study was obtained from the *Institutional Ethics Committee of Manipal University Jaipur, Rajasthan, India.*

The committee reviewed and approved all study procedures in accordance with the ethical standards outlined in the *Declaration of Helsinki (2013 revision).* Written informed consent was obtained from all interview participants prior to data collection. No minors or vulnerable populations were involved in this study.

## Data Availability

Dataset https://doi.org/10.5281/zenodo.17491768 ( Chakravarty, 2025). Data are available under the terms of the Creative Commons Attribution 4.0 International license (CC-BY 4.0).
