# Supplementary material for: Integrating Industry, Innovation, and Infrastructure for Sustainable Development: A TCCM Framework Analysis of SDG 9 Implementation
Source: F1000Res. 2026 Mar 26;15:439. [Version 1] doi: 10.12688/f1000research.172412.1 (PMC13507844; doi:10.12688/f1000research.172412.1)
Supplement: Peer review history

# Reviewer report: Reviewer response for version 1

**DOI:** 10.5256/f1000research.190134.r496364

**Published:** 2026-07-09

**Authors:** Arun Arora

**Affiliations:** 1 Department of Business Studies, Lloyd Business School, Greater Noida, Uttar Pradesh, India

The article is very well written. The outcome of the study addresses the SDG-9 role. Literature review using TCCM framework is systematically and written with logical flow. Data analysis was  done in two phases, which shows the  accuracy of  outcomes. Implications of  the  study very well explained. I am recommending this article for indexing.

Are the rationale for, and objectives of, the Systematic Review clearly stated?

Yes

Is the statistical analysis and its interpretation appropriate?

Yes

If this is a Living Systematic Review, is the ‘living’ method appropriate and is the search schedule clearly defined and justified? (‘Living Systematic Review’ or a variation of this term should be included in the title.)

Yes

Are sufficient details of the methods and analysis provided to allow replication by others?

Yes

Are the conclusions drawn adequately supported by the results presented in the review?

Yes

Reviewer Expertise:

Operations , Supply chain management, Multi-disciplinary research

I confirm that I have read this submission and believe that I have an appropriate level of expertise to confirm that it is of an acceptable scientific standard.

# Response

**Published:** 2026-07-11

**Authors:** Dr.Shilpi Chakravarty

**Affiliations:** CDOE, Manipal University Jaipur, Jaipur, Rajasthan, India

Thank you so much sir for your valuable comments.

# Reviewer report: Reviewer response for version 1

**DOI:** 10.5256/f1000research.190134.r496362

**Published:** 2026-06-26

**Authors:** Praveen Gujjar

**Affiliations:** 1 JAIN (Deemed-to-be University), Bengaluru, Karnataka, India

Article is well written and results are satisfactory. Primary Data are collected through semi-structured interviews with stakeholders—policymakers, industry leaders, and infrastructure experts—in three purposively selected countries representing high, middle, and low SDG 9 performance. Quantitative analysis demonstrates that governance quality, research and development investment, and digital connectivity significantly influence infrastructure and innovation outcomes.

Are the rationale for, and objectives of, the Systematic Review clearly stated?

Yes

Is the statistical analysis and its interpretation appropriate?

Yes

If this is a Living Systematic Review, is the ‘living’ method appropriate and is the search schedule clearly defined and justified? (‘Living Systematic Review’ or a variation of this term should be included in the title.)

Yes

Are sufficient details of the methods and analysis provided to allow replication by others?

Yes

Are the conclusions drawn adequately supported by the results presented in the review?

Yes

Reviewer Expertise:

Data Analytics

I confirm that I have read this submission and believe that I have an appropriate level of expertise to confirm that it is of an acceptable scientific standard.

## References

[1] : Assessing regional implementation of Sustainable Development Goal 9 “Build resilient infrastructure, promote sustainable industrialization and foster innovation” in Poland. *Technological Forecasting and Social Change* .2023;195: 10.1016/j.techfore.2023.122773 10.1016/j.techfore.2023.122773

# Response

**Published:** 2026-06-27

**Authors:** Dr.Shilpi Chakravarty

**Affiliations:** CDOE, Manipal University Jaipur, Jaipur, Rajasthan, India

Thank you so much sir, for your expert comments...
